# The Biological Role of Sponge Circular RNAs in Gastric Cancer: Main Players or Coadjuvants?

**DOI:** 10.3390/cancers12071982

**Published:** 2020-07-21

**Authors:** Adenilson Leão Pereira, Leandro Magalhães, Rafael Pompeu Pantoja, Gilderlanio Araújo, Ândrea Ribeiro-dos-Santos, Amanda Ferreira Vidal

**Affiliations:** 1 Faculty of Medicine, Federal University of Pará, Altamira 68371-163, Brazil; adenilson.leao@hotmail.com; 2 Research Center on Oncology, Graduate Program of Oncology and Medical Science, Federal University of Pará, Belém 66073-000, Brazil; akelyufpa@gmail.com; 3 Laboratory of Human and Medical Genetics, Institute of Biological Sciences, Graduate Program of Genetics and Molecular Biology, Federal University of Pará, Belém 66075-110, Brazil; leandromag@me.com (L.M.); rafaelpompeu988@gmail.com (R.P.P.); gilderlanio@gmail.com (G.A.)

**Keywords:** sponge circRNA, circHIPK3, circPVT1, circNF1, ciRS-7, circ_0000096, gastric carcinogenesis, gastrointestinal tumors

## Abstract

Circular RNAs (circRNAs) are a new class of long noncoding RNAs able to perform multiple functions, including sponging microRNAs (miRNAs) and RNA-Binding Proteins (RBPs). They play an important role in gastric carcinogenesis, but its involvement during gastric cancer (GC) development and progression are not well understood. We gathered miRNA and/or RBPs sponge circRNAs present in GC, and accessed their biological roles through functional enrichment of their target genes or ligand RBPs. We identified 54 sponge circRNAs in GC that are able to sponge 51 miRNAs and 103 RBPs. Then, we evaluated their host gene expression using The Cancer Genome Atlas (TCGA) database and observed that *COL1A2* is the most overexpressed gene, which may be due to circHIPK3/miR-29b-c/*COL1A2* axis dysregulation. We identified 27 GC-related pathways that may be affected mainly by circPVT1, circHIPK3 and circNF1. Our results indicate that circHIPK3/miR-107/*BDNF*/*LIN28* axis may mediate chemoresistance in GC, and that circPVT1, circHIPK3, circNF1, ciRS-7 and circ_0000096 appear to be involved in gastrointestinal cancer development. Lastly, circHIPK3, circNRIP1 and circSMARCA5 were identified in different ethnic populations and may be ubiquitous modulators of gastric carcinogenesis. Overall, the studied sponge circRNAs are part of a complex RBP-circRNA-miRNA-mRNA interaction network, and are involved in the establishment, chemoresistance and progression of GC.

## 1. Introduction

Gastric cancer (GC) is the fifth most frequently diagnosed cancer and the third leading cancer-related deaths worldwide [1]. It is an aggressive disease, whose five-year survival rate is high in some countries like Japan (60%) and South Korea (69%); however, in regions such as South and North America and Europe, this rate does not exceed 30% [2].

Genomic studies have uncovered key changes that result in the development of GC and, the most studied and characterized epigenetic alteration is the tumor-associated DNA methylation profile [3,4]. However, noncoding RNAs (ncRNAs), such as microRNAs (miRNAs) [5] and circular RNAs (circRNAs) [6], are also dysregulated during this disease onset and progression.

CircRNAs are a new class of long noncoding RNAs that have their 5′ and 3′ ends covalently joined [7,8]. Current knowledge of circRNAs suggests that they can perform multiple functions, such as: sponging miRNAs, transcription and splicing modulators and RNA-Binding Proteins (RBPs, e.g., AGO2 and RNA Pol II). They can also act as templates for protein translation and mediate immune responses [7,8]. Dysregulation in their role as miRNA-sponge has been reported in several types of gastrointestinal malignancies, including hepatocellular carcinoma [9,10,11], pancreatic [10,12], colorectal [10,11,13] and gastric cancer [14,15,16,17,18,19,20,21,22,23,24,25,26,27,28,29,30,31,32,33,34,35,36,37,38,39,40,41,42,43,44,45,46,47,48,49,50,51,52,53,54,55,56,57,58,59,60,61,62,63,64,65,66,67,68,69].

A large number of studies have demonstrated the role of circRNAs as miRNA-sponges in GC [14,15,16,17,18,19,20,21,22,23,24,25,26,27,28,29,30,31,32,33,34,35,36,37,38,39,40,41,42,43,44,45,46,47,48,49,50,51,52,53,54,55,56,57,58,59,60,61,62,63,64,65,66,67,68,69]. For example, it has been shown that alterations in circLARP4/miR-424/LATS1 [57], circRNA_100269/miR-630 [59], circPVT1/miR-125b/E2F2 [14] and circ_0000096/miR-224-5p/miR-200a [58] axes can stimulate proliferation, migration and invasion of GC cells. They were also correlated with staging, poor survival and recurrence of this type of tumor. 

More recently, it has been found that circHIPK3/miR-107/BDNF imbalance leads to proliferation and migration of GC cells, and circHIPK3 has been suggested as a potential prognostic biomarker in GC [17]. Additionally, circBANP/let-7a axis imbalance can lead to tumor progression by affecting FZD5/Wnt/ß-catenin signaling [54]. 

circRNAs can also sponge RBPs in GC and can negatively affect the activity of the anchored protein or favor the expression and/or activity of others. For instance, circ-DONSON has been shown to interact directly with proteins from the NURF chromatin remodeling complex to regulate the transcription of *SOX4* oncogene and promote gastric tumorigenesis [70]. On the other hand, circFAT1(e2) is able to bind directly to Y-box binding protein-1 (YBX1) and block tumor-promoting abilities of GC cells [69]. Although studies have demonstrated a relationship between sponge circRNAs and GC, their biological role is still not well understood, and a broader and more integrated scenario of these molecules in gastric carcinogenesis remains to be outlined, being important for future translational applications. 

In this study, we gathered experimentally validated sponge circRNAs in GC, in order to understand and to have a broader view of their significance during the development of this type of tumor. Hence, we evaluated the biological roles of these circRNAs, considering their associated RBPs and/or the target genes of their sponged miRNAs, and thus, identified their involvement in GC-related pathways. Our results indicate that the sponge circRNAs in GC are part of a complex RBP-circRNA-miRNA-mRNA interaction network, and may be involved in the establishment, chemoresistance and progression of the tumor. 

## 2. Results

**Figure 1:**
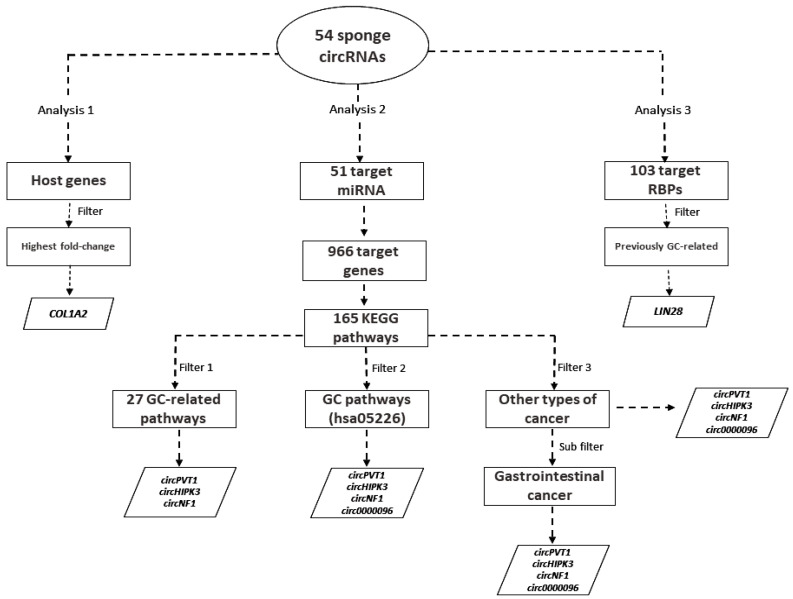
Flowchart showing an overview of our study design and results. After selecting the 54 sponge circRNAs, we divided our study by analyzing their host genes (Analysis 1), target miRNAs (Analysis 2) and target RNA-Binding Proteins (RBPs, Analysis 3). Each analysis was performed using different filters.

Our search identified 56 papers reporting 54 dysregulated sponge circRNAs in GC, of which 38 were upregulated and 16 were downregulated (Table 1). We explored our results by analyzing them in three different ways (Figure 1). Firstly, we investigated the expression and potential roles of the sponge circRNAs’ host genes; then, we analyzed the sponge circRNAs’ target miRNAs and estimated their roles by drawing circRNA-miRNA-mRNA interaction networks; lastly, we focused at the sponged RBPs and their biological roles.

### 2.1. Host Genes Expression Profile in GC

We used GC samples from The Cancer Genome Atlas (TCGA) to access the expression of the sponge circRNAs’ host genes. We found 38 differentially expressed host genes in GC (37 upregulated and one downregulated) when compared to the adjacent tissue (Table S1). Ten genes (*COL1A2*, *FAT1*, *NF1*, *FN1*, *NOTCH1*, *OSBPL10*, *PDSS1*, *PTK2*, *RANGAP1* and *SKA3*) presented |fold change|>2, being *COL1A2*, *PVT1*, *RBMS3* and *SERPINB5* the ones that showed expression levels higher than three-fold in GC (Table S1). In general, circRNAs and their host genes presented similar expression patterns.

### 2.2. Dysregulated Sponge Circular RNAs in GC

The 54 circRNAs are able to sponge 51 different miRNAs (Table 1). These studies validated their findings using sensitive and reliable methodologies, such as RT-qPCR, Western blot, cell and/or luciferase assay report.

In general, we observed 46 circRNAs sponging a single microRNA and eight circRNAs regulating at least two microRNAs simultaneously (Table 1). On the other hand, 10 miRNAs can be sponged by more than one circRNA (miR-107 (circHIPK3 and circ-ZFR); miR-124-3p (circPVT1 and circHIPK3); miR-149 (circ_NRIP1 and hsa_circ_0017728); miR-637 (circ_ERBB2 and circ-NOTCH1); miR-130a-3p (circGRAMD1B and circ-ZFR); miR-182-5p (circ-SFMBT2 and circFN1); miR-1256 (circHECTD1 and circ-DCAF6); miR-503 (circ_ERBB2 and hsa_circ_0000267); miR-145 (circ-PRMT5, circDUSP16 and circMAN2B2); miR-375 (hsa_circ_0008035, circ-SERPINE2 and circUBA1); Table 1).

### 2.3. Functional Enrichment of the Sponged miRNAs’ Target Genes

To identify the experimentally validated target genes of the 51 sponged miRNAs, we submitted them to the miRTargetLink Human online tool. The query showed that of the 51 miRNAs, 39 miRNAs can regulate 966 experimentally validated genes (Table S2). Of these, 159 genes can be regulated by more than one miRNA and *BCL2*, *CDK6*, *SP1*, *IGF1R*, *VEGFA*, *CCND2*, *HMGA2*, *EGFR*, *CDKN1A*, *MCL1* and *PTEN* genes can be regulated by more than five miRNAs each (Table S3). Twelve miRNAs (miR-214-5p, miR-367-5p, miR-369-3p, miR-449c-5p, miR-503-3p, miR-526b, miR-877-3p, miR-1227-5p, miR-1231, miR-1304, miR-1306-3p and miR-4319) did not show experimentally validated target genes according to miRTargetLink Human tool (Table S2). 

**Figure 2:**
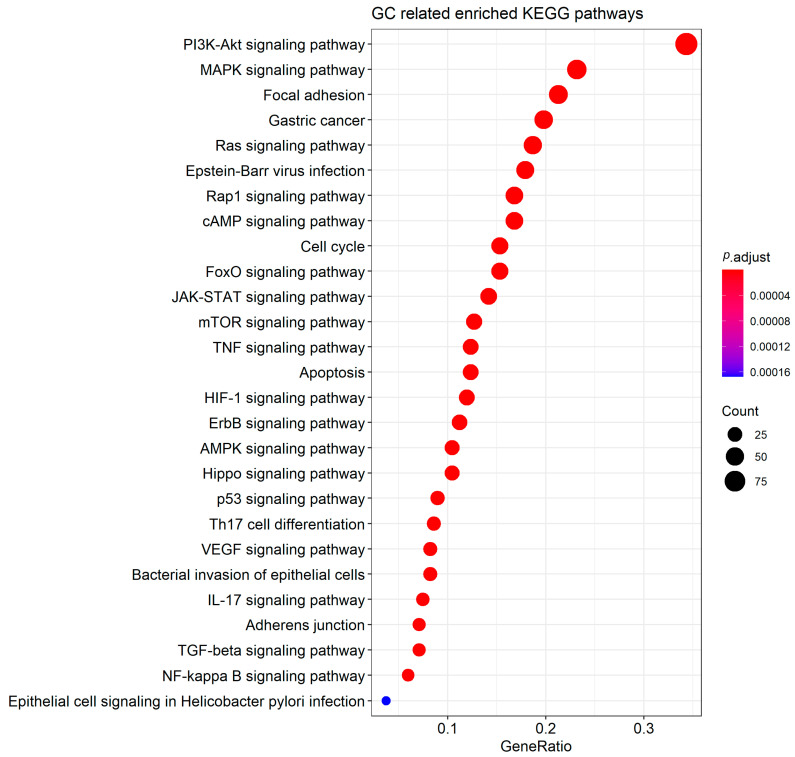
Gastric cancer (GC)-related Kyoto Encyclopedia of Genes and Genomes (KEGG) pathways in which the sponged miRNAs’ target genes are potentially involved.

Functional enrichment analysis showed that these genes are involved in the regulation of 165 Kyoto Encyclopedia of Genes and Genomes (KEGG) pathways (Table S4). Of these, we highlight 27 biological pathways that are closely related to the development of GC (e.g., bacterial invasion of epithelial cells, cell cycle, Epstein–Barr virus infection, epithelial cell signaling in *Helicobacter pylori* infection, etc.; Figure 2). 

By performing differential expression analysis of the 966 miRNAs’ target genes using the TCGA database, we found 45 DE genes (*p* ≤ 0.05 and |fold change| ≥1.5, Table S5). These 45 genes may have a greater impact on gastric cancer and should be further explored. However, we would like to emphasize that an integrative analysis would become more accurate using the same cohort to access mRNA, miRNA and circRNA global expression. By this approach, the identification of hub ncRNAs and genes involved in gastric carcinogenesis would be more precise.

### 2.4. Biological Pathways and the Potential Roles of the Sponge circRNAs in GC

Of the 966 target genes used during functional enrichment analysis, 271 are involved in 27 biological pathways directly associated with GC (Figure 1; Table S6). circPVT1, circHIPK3 and circNF1 may interfere and regulate the expression of the largest number of genes associated with these pathways (107, 103 and 43 genes, respectively; Figure S1). Other circRNAs, such as circLARP4, hsa_circ_0008035, circ_0000096, circ-ZFR, ciRS-7, circ-SFMBT2, hsa_circ_0027599, ciRS-133 and circ-EIF4G3, can regulate the expression of more than 10 genes each, suggesting they have an important role in GC (Table S6). circDLST, circOSBPL10, circNHSL1, circHECTD1, circRHOBTB3, hsa_circ_0001368 and circFAT1(e2) regulate one gene each (Table S6).

According to the KEGG database, the GC pathway (hsa05226) is composed of 147 main genes, of which 53 can be regulated by 24 miRNAs evaluated in this study (Tables S4 and S7). Through the mRNA-miRNA-circRNA interaction, we identified 23 sponge circRNAs that can regulate these miRNAs and may affect the expression of their target genes directly involved in the gastric cancer pathway (Table S7). circNF1, circHIPK3, circPVT1 and circ_0000096 are the ones that can regulate the highest number of genes present in this pathway. Meanwhile, hsa_circ_100269, circ-CEP85L, circSMARCA5, circRHOBTB3, circRBMS3, circOSBPL10 and circAKT3 regulate one gene each (Table S7). *BCL2* gene can be regulated by the greatest number of miRNAs (10 target miRNAs) and, consequently, the gene associated with the highest number of sponge circRNAs (13 circRNAs, Table S7).

### 2.5. Biological Pathways and the Potential Roles of the Sponge circRNAs in Other Cancers

**Figure 3:**
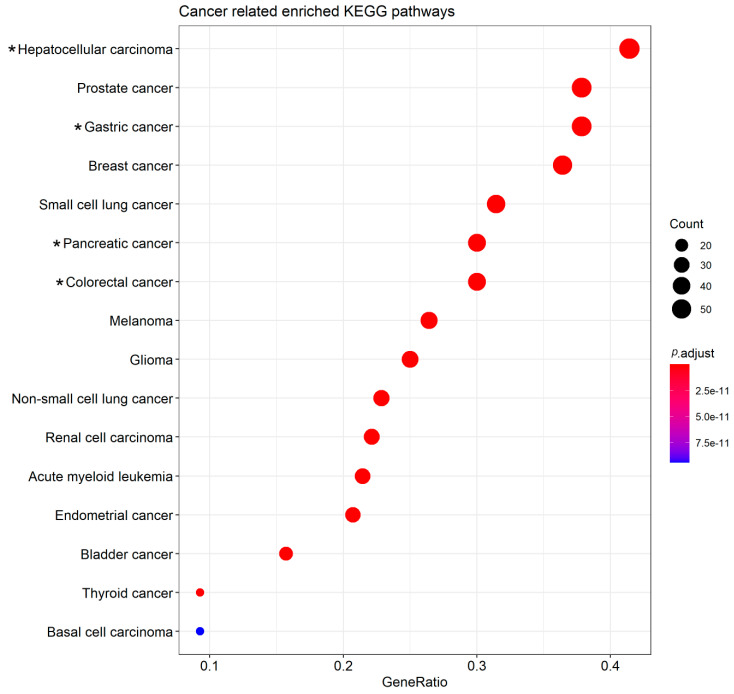
Cancer KEGG pathways in which the sponged miRNAs’ target genes are potentially involved. (*) Gastrointestinal tumors.

Among the 165 biological pathways, we also observed that the circRNA-miRNA-mRNA interaction may be involved in 16 different types of tumors, of which four are gastrointestinal tumors (Figure 3; Table S8). So, we evaluated the types of tumors in which the target genes could be associated (consequently, the sponge circRNAs and their target miRNAs too). We identified that these 16 different types of tumors can be affected by the dysregulated expression of 140 sponged miRNAs’ target genes (Figure 3; Table S8). We also noticed that 51 of them are shared by at least two different tumors of the gastrointestinal tract and can be regulated by 25 miRNAs and 28 sponge circRNAs (Table S8). circHIPK3, circNF1, circ_0000096 and circPVT1 can regulate the highest number of genes (≥11 genes each); while circAKT3, circ-CEP85L, circOSBPL10, circRHOBTB3, circSMARCA5 and hsa_circ_100269 regulate one gene each (Table S8). Twenty-five genes were associated with the four types of gastrointestinal tumors, and can be regulated by 18 miRNAs and 17 circRNAs sponge (Table S8). Of these, *CDKN1A*, *EGFR*, *SMAD4* and *TP53* genes can be regulated by four to five miRNAs each, and by six to eight sponge circRNAs each one (Table S8).

### 2.6. Functional and Enrichment Analysis of RBPs

**Figure 4:**
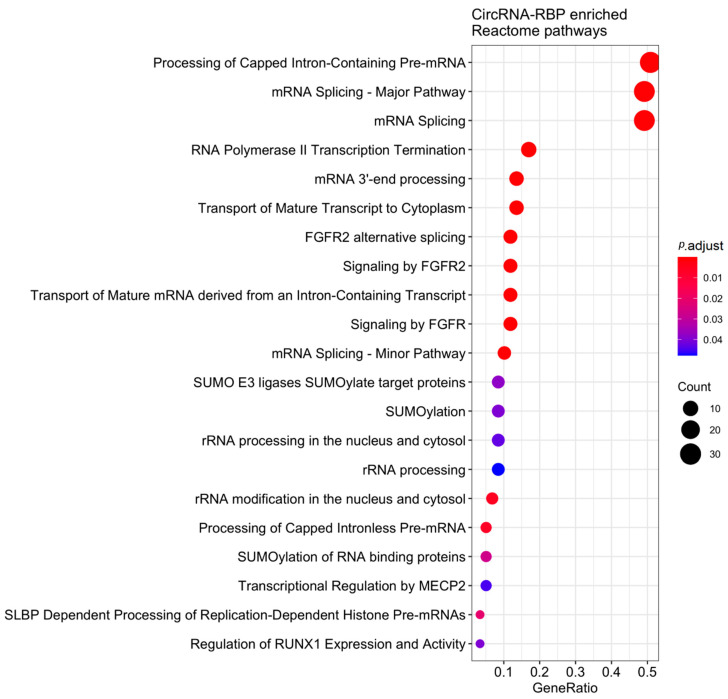
Reactome pathways for which the sponge circRNAs’ target RBPs are enriched.

We also analyzed the existence of RBP sites in the sponge circRNAs. We observed that 13 of the 54 studied circRNAs have RBP anchoring sites (circHIPK3, circPVT1, circFAT1(e2), circAKT3, ciRS-7, circ_SPECC1, circSMARCA5, hsa_circ_0000467, hsa_circ_0000267, hsa_circ_0000673, hsa_circ_0001368, circ_0000096 and hsa_circ_0000993), and that together, they are able to bind to 103 different proteins (Table S9). circFAT1(e2) and hsa_circ_0000673 have the highest number of RBP-binding sites (78 and 76 sites, respectively), while ciRS-7 has 11 sites (Table S9). IGF2BP2 and MOV10 are able to bind to all analyzed circRNAs (Table S10). We highlight LIN28A and LIN28B, which have binding sites in 11 circRNAs (Table S10) and were previously related to the GC [71,72]. Functional enrichment analysis of these proteins revealed that they are involved in 27 different Reactome pathways, and that they have a strong association with RNA metabolism and processing (Figure 4).

## 3. Discussion

Although genomic big data have found key genetic modifications that are capable of leading to GC onset [3,4], studies have elegantly shown that a complex, dysregulated and poorly understood regulatory network that involves ncRNAs, mRNAs and RBPs can induce gastric tumorigenesis.

In this study, we gathered circRNAs that can sponge miRNAs and RBPs, and observed that many of them may break homeostasis and disturb biological pathways in GC. An overview of our analyses and results is presented in Figure 1. For instance, the circHIPK3/miR-124/miR-29b/COL1A1/COL4A1/CDK6 [16], ciRS-7/miR-7/PTEN/PI3K/AKT [18] and other signaling pathways (Table S11) may promote the onset, establishment and progression of the tumor by modulating the cell cycle progression, tumor growth, proliferation, apoptosis, invasion, migration, survival and radiosensitivity, stemness, epithelial–mesenchymal transition (EMT) and metastasis of malignant gastric cells. In addition, dysregulation of these axes has an important clinical impact during the course of the disease, since they are associated with poor prognosis, overall and disease-free survival and clinicopathological characteristics (Table S11).

*COL1A2* was the circRNAs’ host gene with the highest fold change in GC (Table S1). This gene was positively related to clinicopathological characteristics such as tumor size and depth of invasion in GC [73]. Interestingly, circHIPK3 upregulates *COL1A1* and *COL4A1* expression by sponging miR-29b and miR-124, promoting GC progression [16]. Meanwhile, *COL1A2* has been shown to be a direct target of miR-29b and miR-29c [74,75], which are members of the miR-29 family (miR-29a, 29b and 29c) and share a common seed sequence [76]. Since miR-29b and miR-29c are known to be downregulated in GC [77,78], it is possible that the overexpression of *COL1A2* observed in gastric tumor tissue is maintained in part by the disturbance in circHIPK3/miR-29b-c/*COL1A2* axis, leading to gastric cancer progression.

Gene functional enrichment in biological pathways indicates that circRNA/miRNA/mRNA axes can directly affect several pathways (Figure 2) that are known to be dysregulated during gastric carcinogenesis [79,80,81]. We highlight circPVT1, circHIPK3 and circNF1 that have the potential to regulate the expression of the highest number of target genes related to these pathways, suggesting that they are critical modulators in gastric adenocarcinoma. Therefore, these findings indicate that the sponge circRNAs may modulate a greater number of pathways in GC than have been associated so far.

Among the pathways shown in Figure 2, we highlight PI3K/Akt signaling pathway because it has the highest number of sponged miRNAs’ target genes and can be regulated by 28 miRNAs and 33 circRNAs (Tables S4–S6). This pathway can be modulated by different epigenetic mechanisms, such as noncoding RNAs, DNA methylation and chromatin remodeling systems [82,83,84]. Here, we highlight *BCL2*, *CDK4*, *CDK6*, *CDKN1A*, *EGFR*, *ERBB2*, *FGFR1*, *IGF1R*, *ITGB1*, *MYC*, *NRAS*, *KRAS*, *PHLPP1*, *PIK3CA*, *PTEN*, *TP53* and *VEGFA*, genes that are potentially the most affected since they are modulated by the highest number of circRNA/miRNA axes (Table S6). In addition, *AKT1* (circPVT1/miR-125b-5p), *AKT2* (circHIPK3/miR-29b-3p) and *AKT3* (cirNF1/miR-16-5p) may also be under the regulatory influence of these axes (Table S6). Interestingly, many of these genes have been found to be associated with this pathway [82,85] and promoting gastric carcinogenesis [3,4].

**Figure 5:**
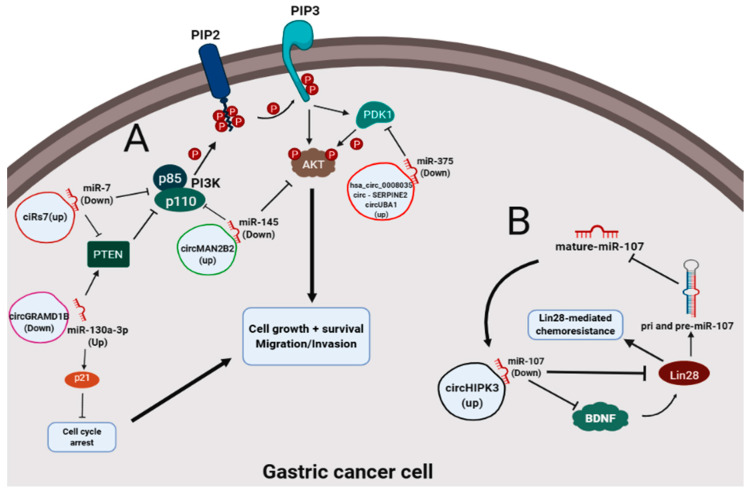
Graphic representation of signaling pathways that promote gastric cancer. (**A**) circRNA-miRNA interactions that affect the PI3K/Akt signaling pathway in gastric cancer. (**B**) circHIPK3/miR-107/Lin28-mediated chemoresistance of gastric cancer cells.

PI3K/Akt signaling pathway regulates metabolism, survival, cell growth, motility and angiogenesis in several tumors [86,87]. In this pathway, there is the recruitment of phosphorylated AKT (pAkt) and PDK1 (a kinase that phosphorylates Akt protein) by PIP3 action (class IA PI3Ks), caused by the downregulation of *PTEN*, a negative regulator of PIP3 and PI3K [79,83]. *AKT* is responsible for activating growth, proliferation and survival signaling [79]. Interestingly, we have found several circRNA-miRNA interactions that are capable of affecting the activity or expression of genes (e.g., *PTEN*, *PI3K*, *AKT*, *p21* and *PDK1*) that are directly involved in PI3K/Akt pathway (Table 2). For instance, circGRAMD1B/miR-130a-3p axis negatively regulates PTEN and p21, allowing higher proliferation, migration and invasion rates of GC cells [62]. Curiously, p21 is a cyclin/cdk inhibitor protein directly associated with the PI3K/Akt pathway [88]. We also highlight the tumor suppressor miR-375 that decreases PDK1 activity in PI3K/Akt signaling [79]. We observed that this miRNA is sponged by three different circRNAs (Table 2), suggesting that they can modulate the miR-375/PDK1/Akt axis to activate cell growth, survival and motility in GC (Table 2, Figure 5A). Therefore, the circRNAs we found may potentially promote gastric carcinogenesis by affecting mainly the PI3K/Akt pathway (Figure 5A).

Regarding the gastric cancer pathway (hsa05226), we noticed that it can be more affected by circNF1, circHIPK3, circPVT1 and circ_0000096, since they can regulate many target genes (Table S7). These circRNAs were also the most critical for other types of gastrointestinal tumors (Table S8). Surprisingly, circHIPK3 and circPVT1 have already been found to be dysregulated in hepatocellular carcinoma, colorectal and pancreatic cancers [9,10,11,12,13]. ciR-7 is another circRNA that is dysregulated in these four types of tumors [11]. Interestingly, circNF1, circHIPK3 and circPVT1 are involved in the 27 GC-related pathways, in the GC pathway itself (hsa05226) and in other cancer-associated pathways, including gastrointestinal ones (Figure 1). Therefore, we suggest that circHIPK3, circPVT1, ciR-7, circNF1 and circ_0000096 may be involved in gastrointestinal carcinogenesis. However, it is necessary to experimentally validate whether circNF1 and circ_0000096 are also dysregulated in hepatocellular carcinoma, colorectal and pancreatic cancer. 

circRNAs can act as an anchoring point for RBPs and this interaction can block these binding protein’s activity [69]. We found that circAKT3, circFAT1(e2), circHIPK3, circPVT1, circSMARCA5, circ_SPECC1, circ_0000096, hsa_circ_0000993, hsa_circ_0000467, hsa_circ_0001368 and hsa_circ_0000673 may bind to LIN28A and LIN28B. High Lin28 protein levels seem to mediate the chemoresistance to drugs such as oxaliplatin, paclitaxel, doxorubicin and fluorouracil in GC by regulating miR-107 expression [71,72,89]. Interestingly, only four circRNAs were overexpressed (circAKT3, circHIPK3, circPVT1 and hsa_circ_0000467) in GC, while the remaining seven were downregulated (Table 1), suggesting that the low expression of these circRNAs may allow the activity of LIN28 in GC to favor chemoresistance.

Although LIN28 can inhibit the maturation of miR-107, this miRNA can also negatively regulate LIN28 [89]. LIN28 is capable of blocking the maturation of a subset of miRNA precursors (i.e., pri- and pre-miRNA) such as let-7, miR-107, miR-143 and miR-200c [90,91]. circHIPK3 also has an inhibitory effect on miR-107 expression by sponging it, which allows brain-derived neurotrophic factor (BDNF) activity [17]. Interestingly, it was observed that BDNF is a positive regulator and increases the activity of LIN28 to act in the regulation of specific miRNAs [91]. In this context, we believe that LIN28 suppresses miR-107 at the beginning of its biogenesis, while circHIPK3 reinforces the repression of this miRNA (those that eventually deviated from the repression via LIN28) in its mature state and enhances LIN28 activity (Figure 5B). Therefore, circHIPK3/miR-107/BDNF/LIN28 axis is an alternative mechanism proposed for LIN28-mediated chemoresistance in GC (Figure 5B) and requires experimental validation.

Lastly, of the 54 circRNAs identified in this study, all were tested in the Asian/Chinese population. However, we observed that circHIPK3, circNRIP1 and circSMARCA5 were also identified in the Brazilian population [6]. The Brazilian population is highly substructured and has a strong genetic mixture, due to the genetic contribution of different parental populations, such as European, African, Asian and Amerindian ones [92,93]. It is known that the genetic/ethnic contribution may influence the expression of many genes in response to diseases and/or infections [94,95]. Although the ethnic factor contributes to the modulation of the expression of some genes, in the case of these three circRNAs this apparently does not occur. Thus, we suggest that these three circRNAs may be key ubiquitous modulators of gastric carcinogenesis.

The sponge function of the circRNAs has been debated since most of them harbor only a few miRNA-binding sites and effective target repression may often require many binding sites [96]. However, the growing number of experimental studies have demonstrated that the circRNAs are able to successfully repress miRNAs by sponging them (Table S11). Our network-based approach provided a valuable and easy way of integrating heterogeneous data, which allowed better recognition of a regulatory module involving RBP-circRNA-miRNA-mRNA interactions in gastric cancer.

In general, comprehensive bioinformatics analysis of regulatory networks has pointed out potential interactions and new biological roles between small and long noncoding RNAs associated with complex diseases, such as gastric and colorectal cancers [97,98,99]. We understand that there are inherent limitations in predictive computational analysis; however, in this study, these limitations were minimized by only using experimentally tested and validated data, in order to avoid biased results. This integrative study, using tested biological interactions and computational analysis, offers a broader picture of the epigenetic complexity that involves RBP-ncRNA-mRNA in gastric tumorigenesis.

## 4. Materials and Methods

### 4.1. Search for the Sponge circRNAs

Sponge circRNAs were extracted from previous studies selected from PubMed and/or Google Scholar repository databases. Only original research articles published until March 2020 were selected. Keywords that relate and/or associate sponge circRNA, miRNA and gastric cancer were used in the search. In both repositories, we used the following entries: “circRNA sponge and Gastric Cancer”, “circRNA sponge in Gastric Cancer” and “circRNA-miRNA in Gastric Cancer”. Only studies that conducted experimental validation (e.g., RT-qPCR, Western blot, cell and/or luciferase reporter assay) of the analyzed sponge circRNA were included in the analyzes. Studies based only on in silico predictions of circRNA-miRNA interaction were excluded. Restrictions by year of publication or language were not applied.

### 4.2. Expression Profile of the Sponge circRNAs’ Host Genes 

To analyze the differential expression of sponge circRNAs’ host genes, we used the “UALCAN: Analyze, Integrate, Discovery” online tool [100] (http://ualcan.path.uab.edu/index.html). This tool is connected to The Cancer Genome Atlas (TCGA) database and provides OMICS expression data of 415 gastric cancer and 34 controls (adjacent to gastric cancer) samples in transcripts per million (TPM). 

### 4.3. Target Gene Identification and Enrichment Analysis 

Target genes of the sponged miRNAs were analyzed using miRTargetLink Human online tool [101] (https://ccb-web.cs.uni-saarland.de/mirtargetlink/). This tool is connected to the miRTarBase database (release 7.0) [102] (http://miRTarBase.cuhk.edu.cn/), which provides information on experimentally validated interaction between the miRNA and its target gene. We only considered target genes that were validated with “strong evidence” (e.g., RT-qPCR, Western blot, cell and/or luciferase reporter assay).

Functional enrichment analysis of the sponged miRNAs’ target genes was performed using Kyoto Encyclopedia of Genes and Genomes (KEGG) and/or Reactome pathways in STRING online tool (v.11.0). In this analysis, we considered the total number of target genes of each miRNA. Graphs were made in R statistical package (Release 3.6.0; https://www.r-project.org/) [103,104]. 

### 4.4. Target RBPs and Functional Analysis

Target RBPs of the sponge circRNAs were accessed using the starBase (v. 2.0) online tool (http://starbase.sysu.edu.cn/starbase2/index.php), which provides large-scale CLIP-Seq data of circRNA-RBP interaction. Its functional analysis was performed using Reactome pathways in STRING online tool (v.11.0). Graphs were made in R statistical package (Release 3.6.0; https://www.r-project.org/) [103,104].

## 5. Conclusions

The functional roles of the sponge circRNAs in the pathophysiology of GC remains poorly understood. However, in this study, we pointed to new directions for understanding this context. Overall, the 54 sponge circRNAs studied are capable of interfering in 27 GC-related biological pathways by modulating the interaction of 51 different miRNAs and their target genes, with a more pronounced effect on PI3K/Akt signaling pathway. *COL1A2* was the host gene with the highest fold change and circHIPK3/miR-29b-c/*COL1A2* axis may be the one that maintains high levels of this gene in GC, favoring the severe clinicopathological characteristics. Thirteen circRNAs may interact with 103 RBPs that are mostly associated with RNA metabolism. Among them, Lin28 stands out for being previously associated with GC. In this context, circHIPK3 may control the balance between LIN28/miR-107 to favor the activity of Lin28 in a negative feedback loop, promoting chemoresistance of GC cells. circHIPK3, circNRIP1 and circSMARCA5 are involved with GC in different ethnic populations and may be ubiquitous regulators of this type of tumor. Finally, circHIPK3, circPVT1, ciR-7, circNF1 and circ_0000096 are potentially promoting gastrointestinal tumors. Therefore, the sponge circRNAs are favoring gastric carcinogenesis by affecting complex mRNA-miRNA-circRNA-RBP interaction networks, and future studies are required to validate their potential as therapeutic targets for this type of tumor. In this context, the identified sponge circRNAs appear to play a central role in the background of gastric tumors and may be considered major players in tumorigenesis.

## Figures and Tables

**Table 1:** Description of the sponge circular RNAs (circRNAs) in gastric cancer and their target microRNAs (miRNAs).

Sponge circRNA	Expression	Host Gene	Expression	Sponged miRNA	Ref.
hsa_circ_0001821 (circPVT1)	UP	*PVT1*	UP *	miR-124-3p; miR-125b-5p	[14,15]
hsa_circ_0000284 (circHIPK3)	UP	*HIPK3*	UP *	miR-124-3p; miR-29b-3p; miR-107	[16,17]
hsa_circ_0001946 (ciRS-7)	UP	*CDR1*	DOWN *	miR-7-5p	[18]
hsa_circ_0064644 (circRBMS3)	UP	*RBMS3*	DOWN *	miR-153	[19]
hsa_circ_0056618	UP	*SPOPL, UP*	UP	miR-206	[20]
hsa_circ_0027599	DOWN	*PHLDA1*	UP	miR-101-3p.1	[21]
hsa_circ_0007766 (circ_ERBB2)	UP	*ERBB2*	UP	miR-503; miR-637	[22]
hsa_circ_0000267	UP	*FAM53B*	UP	miR-503-5p	[23]
hsa_circ_0089548 (circ-NOTCH1)	UP	*NOTCH1*	UP	miR-637	[24]
hsa_circ_0089547 (circ-NOTCH1)	UP	*NOTCH1*	UP	miR-449c-5p	[25]
hsa_circ_0067997	UP	*FNDC3B*	UP	miR-515-5p	[26]
hsa_circ_0004771 (circ_NRIP1)	UP	*NRIP1*	UP	miR-149-5p	[27]
hsa_circ_0017728	UP	*DHTKD1*	UP	miR-149	[28]
hsa_circ_0081143	UP	*COL1A2*	UP	miR-646	[29]
hsa_circ_0042881 (circNF1)	UP	*NF1*	UP	miR-16	[30]
hsa_circ_0032627 (circDLST)	UP	*DLST*	UP	miR-502-5p	[31]
hsa_circ_0093398 (circPDSS1)	UP	*PDSS1*	UP	miR-186-5p	[32]
hsa_circ_0010522 (ciRS-133)	UP	*RAP1GAP*	DOWN *	miR-133	[33]
hsa_circ_0092303 (circCACTIN)	UP	*C19orf29*	UP	miR-331-3p	[34]
hsa_circ_0008035	UP	*EXT1*	UP	miR-375	[35]
hsa_circ_0008365 (circ-SERPINE2)	UP	*SERPINE2*	UP	miR-375	[36]
hsa_circ_0090410 (circUBA1)	UP	*UBA1*	UP	miR-375	[37]
hsa_circ_0047905	UP	*SERPINB5*	UP *	miR-4516; miR-1227-5p	[38]
hsa_circ_0005075 (circ-EIF4G3)	UP	*EIF4G3*	UP	miR-335	[39]
hsa_circ_0008549 (circOSBPL10)	UP	*OSBPL10*	UP	miR-136-5p	[40]
hsa_circ_0000199 (circAKT3)	UP	*AKT3*	UP *	miR-198	[41]
hsa_circ_0006835 (circNHSL1)	UP	*NHSL1*	UP *	miR-1306-3p	[42]
hsa_circHECTD1	UP	*HECTD1*	UP *	miR-1256	[43]
hsa_circ_0009109 (circ-DCAF6)	UP	*DCAF6*	UP	miR-1231; miR-1256	[44]
hsa_circ_0031250 (circ-PRMT5)	UP	*PRMT5*	UP	miR-145; miR-1304	[45]
hsa_circ_0003855 (circDUSP16)	UP	*DUSP16*	UP	miR-145-5p	[46]
hsa_circ_0069086 (circMAN2B2)	UP	*MAN2B2*	UP	miR-145	[47]
hsa_circ_0017639 (circ-SFMBT2)	UP	*SFMBT2*	UP	miR-182-5p	[48]
hsa_circ_0058147 (circFN1)	UP	*FN1*	UP	miR-182-5p	[49]
hsa_circ_0000467	UP	*SKA3*	UP	miR-326- 3p	[50]
hsa_circ_0003221 (circPTK2)	UP	*PTK2*	UP	miR-369-3p	[51]
hsa_circ_0063526 (circ-RanGAP1)	UP	*RANGAP1*	UP	miR-877-3p	[52]
hsa_circ_0066436 (circATXN7)	UP	*ATXN7*	UP	miR-4319	[53]
hsa_circ_0040809 (circBANP)	UP	*BANP*	UP	miR-let-7a	[54]
hsa_circ_0077736 (circ-CEP85L)	DOWN	*CEP85L (C6ORF204)*	DOWN *	miR-942-5p	[55]
hsa_circ_00074444 (circRHOBTB3)	DOWN	*RHOBTB3*	UP *	miR-654-3p	[56]
hsa_circ_101057 (circLARP4)	DOWN	*LARP4*	UP	miR-424-5p	[57]
hsa_circ_0000096	DOWN	*HIAT1*	UP	miR-224-5p; miR-200a-3p	[58]
hsa_circ_0013048 (hsa_circ_100269)	DOWN	*LPHN2*	UP	miR-630	[59]
hsa_circ_0000673	DOWN	*RSL1D1*	UP	miR-532-5p	[60]
hsa_circ_0072088 (circ-ZFR)	DOWN	*ZFR*	UP	miR-107; miR-130a-3p	[61]
hsa_circ_0004798 (circGRAMD1B)	DOWN	*GRAMD1B*	DOWN *	miR-130a-3p	[62]
hsa_circ_0002320 (circYAP)	DOWN	*YAP1*	UP	miR-367-5p	[63]
hsa_circ_0000745 (circ_SPECC1)	DOWN	*SPECC1 (CYTSB)*	UP	miR-526b	[64]
hsa_circ_0000993	DOWN	*ATL2*	DOWN *	miR-214-5p	[65]
hsa_circ_0001445 (circSMARCA5)	DOWN	*SMARCA5*	UP	miR-346	[66]
hsa_circ_0021977 (circPSMC3)	DOWN	*PSMC3*	UP	miR-296-5p	[67]
hsa_circ_0001368	DOWN	*KLHL24*	UP	miR-6506-5p	[68]
hsa_circ_0001461 (circFAT1(e2))	DOWN	*FAT1*	UP	miR-548g	[69]

(*) Statistically not significant. UP: Upregulated; DOWN: Downregulated; Ref.: References.

**Table 2:** circRNA-miRNA interactions that affect PI3K/Akt signaling pathway in gastric cancer.

circRNA/miRNA Interaction [Ref.]	Effect in Gene and/or Protein [Ref.]	Effect in Gastric Cancer [Ref.]
ciRS-7↑/miR-7↓ [18]	*PTEN*↓, *PI3K*↑ and pAkt↑ [18]	Apoptosis inhibition, migration stimulus and poor clinicopathological and survival [18]
circNRIP1↑/miR-149-5p↓ [27]	*AKT1*↑ [27]	Cell proliferation, migration and invasion and growth of tumor [27]
circGRAMD1B↓/miR-130a-3p↑ [62]	*PTEN*↓ and *p21*↓ [62]	Cell proliferation, migration and invasion [62]
circRNA0047905↑ [38]	*AKT1*/*CREB*↑ [38]	Cell proliferation and tumor progression [38]
circMAN2B2↑/miR-145↓ [47]	pPI3K↑ and pAkt ↑ [47]	Cell growth and migration [47]
hsa_circ_0008035↑/miR-375↓ [35]	*PDK1*↑ *	Increased of pAkt (Proliferation, migration and invasion) *
circ-SERPINE2↑/miR-375↓ [36]	*PDK1*↑ *	
circUBA1↑/miR-375↓ [37]	*PDK1*↑ *	

(*) Mechanism proposed in this study. ↑: Upregulated; ↓: Downregulated; Ref.: References.

## Supplementary Materials

The following are available online at https://www.mdpi.com/2072-6694/12/7/1982/s1, Figure S1: Most important circRNAs to 27 GC-related pathways, Table S1: Host genes expression in GC from TCGA database obtained by UALCAM online tool, Table S2: Sponged miRNAs target genes provided by miRTargetLink Human - Only target genes valued by strong evidence, Table S3: List of miRNAs and target genes, Table S4: Functional analysis of sponged miRNAs’ target genes, Table S5: Genes differentially expressed in GC, Table S6: Intersection among the lists of genes of the 27 GC-related KEGG pathways and the sponged miRNAs’ target genes, Table S7: Genes involved in gastric cancer pathway (hsa05226) and their associated mIRNAs and sponge circRNAs, Table S8: Intersection between the lists of genes of the 16 cancer KEGG pathways and the sponged miRNAs’ target genes, Table S9: List of the target RBPs, Table S10: Target RBPs and their sponge circRNAs, Table S11: Clinical importance and main effects of circRNA-miRNA-mRNA interaction in GC.
