# Supplementary figures and images for: The Biological Role of Sponge Circular RNAs in Gastric Cancer: Main Players or Coadjuvants?
Source: Cancers (Basel). 2020 Jul 21;12(7):1982. doi: 10.3390/cancers12071982 (PMC7409348; doi:10.3390/cancers12071982)

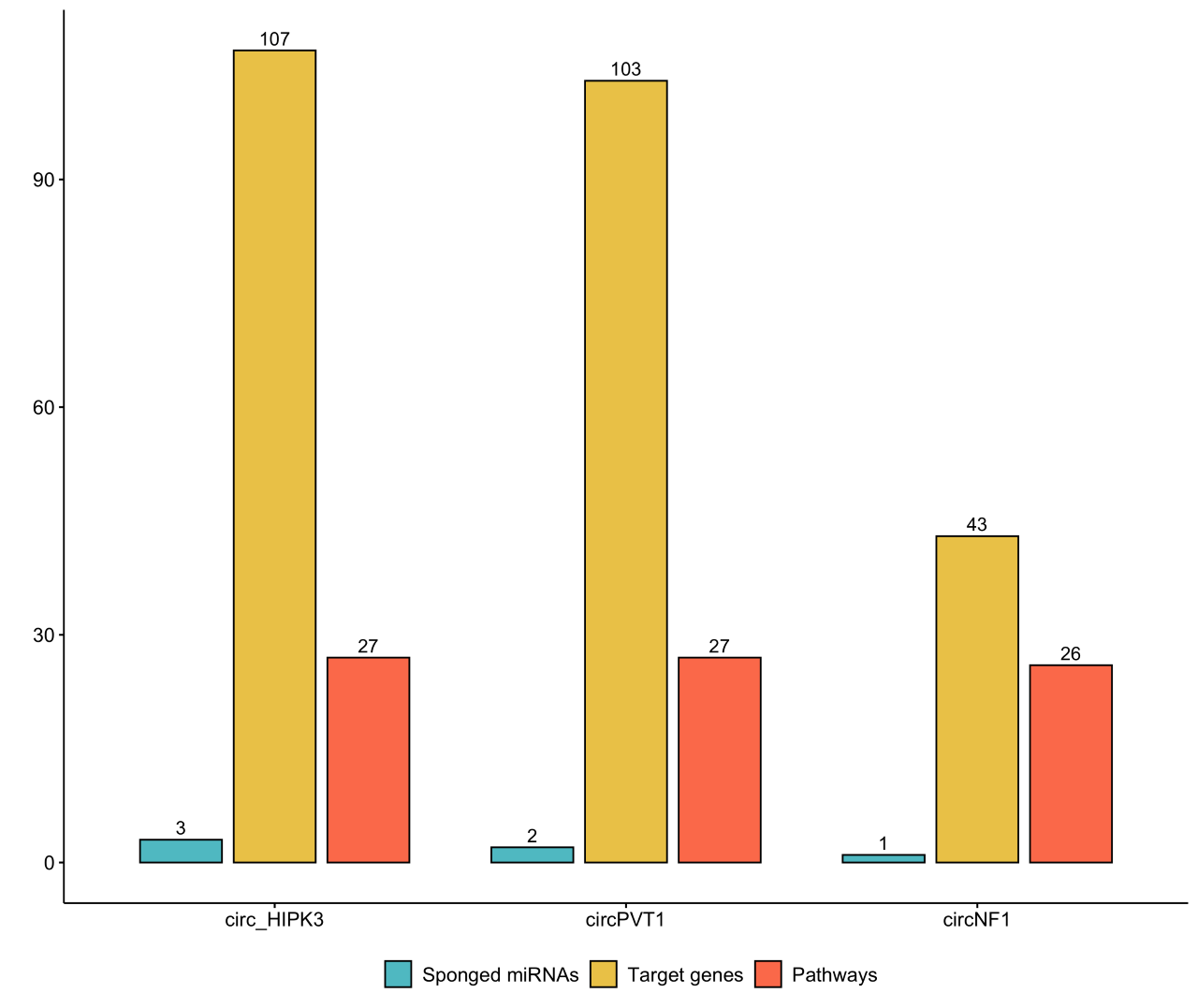

Supplement: Supplementary file 1 [file cancers-12-01982-s001.zip › Supplementary data/Supplementary Figure S1.png]
